# Overexpression of RLIP76 Required for Proliferation in Meningioma Is Associated with Recurrence

**DOI:** 10.1371/journal.pone.0125661

**Published:** 2015-05-20

**Authors:** Song-Yuan Fan, Jian-Dong Jiang, Jun Qian, Yi-Cheng Lu, Guo-Han Hu, Chun Luo, Wei-Dong Hou, Qi Wang

**Affiliations:** 1 Department of Neurosurgery, PLA No.322 hospital, 2 Yunzhong Road, Shanxi 03700,China; 2 Department of Neurosurgery, the 174th hospital of PLA (Chenggong Hospital, Xiamen University), Xiamen 361003, China; 3 Department of Neurosurgery, Changzheng Hospital, Second Military Medical University, 415 Fengyang Road, Shanghai 200003, China; Weizmann Institute of Science, ISRAEL

## Abstract

The GTPase-activating protein RLIP76 is overexpressed in and correlates with the pathological grade of many malignant tumor cells. But the potential correlation between RLIP76 and clinical outcomes in patients with meningioma remains unknown. In this study, we examined the expression of RLIP76 in meningioma and correlated the RLIP76 expression to the patient outcome. RLIP76 expression in tumor tissues was examined with immunohistochemistry, quantitative reverse-transcription polymerase chain reaction(RT-PCR) and Western-blot. Immunohistochemistry showed an increased RLIP76 immunostaining score in anaplastic and atypical meningiomas versus classical meningiomas. Statistical analyses revealed that RLIP76 immunostaining positively correlated with immunostaining for Ki-67, a nuclear protein highly expressed in proliferating cells(r=0.29, *p*=0.034 by Spearman's correlation coefficient). Clinicopathological evaluation suggested that RLIP76 expression be associated with tumor grade and recurrence(*P*<0.05). Univariate and Cox analysis indicated that RLIP76 was an independent prognostic factor for tumor recurrence. Furthermore, the human malignant meningioma cell lines IOMM-Lee and CH157-MN stably transfected with short hairpin RNA (siRNA) targeting RLIP76 were then examined by in vitro growth assays, and apoptosis assays. RLIP76 knockdown in IOMM-Lee and CH157-MN cells inhibited cell proliferation and induced apoptosis. Western blot analysis revealed that cells underexpressing RLIP76 exhibited decreased B-cell lymphoma-2(Bcl-2) expression but increased apoptosis effector caspase-3 expression. These findings demonstrate that high RLIP76 expression is associated with a poor outcome of meningioma and may provide a new gene therapy approach for patients with malignant meningiomas.

## Introduction

Meningioma is a common primary neoplasm of the central nervous system in adults and it accounts for approximately 24–30% of primary intracranial neoplasm [1, 2]. According to the 2007 WHO Classification of Tumors of the Central Nervous System, meningothelial tumors were classified into three grades based on their biologic potential—Meningioma(WHO I), atypical meningioma(WHO II) and anaplastic meningioma(WHO III). Although relatively infrequent, malignant meningiomas are associated with poorer prognosis in contrast with benign meningiomas [3, 4]. Even with aggressive interventions, including surgery, radiotherapy and other therapeutic modalities, it does not ensure a cure especially for those recurrent and unresectable meningiomas [5]. Over the last years, there has been an increasingly number of studies on the molecular genetics of meningiomas [4]. These efforts aim to target new molecular markers and achieve earlier diagnosis and better treatment.

RLIP76, a GTPase-activating protein, is also cloned as a Ral-effector protein linking Ral-GTPase to Rho pathway [6, 7]. It functions as an intermediate signaling molecule that regulates mitosis [8], apoptosis [9] and endocytosis [10]. Recently, a large number of studies have documented hyperactivated RLIP76 signaling in a variety of malignancies, including lung, colon, prostate, and kidney cancer compared with their corresponding normal tissues [11–14]. In addition, genetically engineered mice have highlighted the important role of RLIP76 activation in tumor growth and resistance to chemotherapy [15, 16]. We previously demonstrated that RLIP76 overexpression was also observed in gliomas, indicating the therapeutic effectiveness of RLIP76 inhibitors in glioams [17]. Furthermore, accumulating evidence suggests that overexpression of RLIP76 is strong positively correlated with advanced tumor grade and negatively correlated with survival in mammary tumors [14, 17]. However, expression pattern and biological functions of RLIP76 in meningiomas are unknown.

In this study, we detected RLIP76 expression in meningiomas and decreased RLIP76 expression in IOMM-Lee and CH157 cells to clarify the role of RLIP76 on cell apoptosis and proliferation in vitro. Since RLIP76 plays a requisite role in the development of tumor, a clear understanding of the interaction at the molecular level could potentially open up new therapeutic avenues for the treatment of malignant meningiomas.

## Materials and Methods

### Tissue samples

The study protocol and acquisition of tissue specimens were approved by the Specialty Committee on Ethics of Biomedicine Research, Second Military Medical University. Ethics Committee of Second Military Medical University specifically approved that no informed consent was required because data were analyzed anonymously and the specific samples used in this study have been described in our previous study [18]. Human tissue acquisition and use in this study complied with the National Regulations on the Use of Clinical Samples in China. Tissue specimens were obtained from archived tissue samples from patients with meningiomas who underwent surgical treatment at Changzheng Hospital and PLA NO.322 hospital from January 1999 to December 2011. Meningioma was diagnosed according to the 2007 WHO Classification of Tumors of the Central Nervous System by two experienced pathologists independently. The follow-up was carried out in all patients, with survival time being censored in July 2012. The follow-up was conducted every 6 months by telephone and the end of the follow-up was January 2013. Other treatment options, including adjuvant chemotherapy and radiotherapy, were fully discussed with the patients. The tumor size, location, extent of surgical resection, and time free from recurrence were recorded. Extent of resection was defined in each case in terms of the Simpson grading system. The clinicopathologic characteristics of the patients are summarized in Table 1. The selection criteria were described in detail previously [18]. According to the selection criteria, 106 tissue samples (classical meningiomas-WHO grade I, n = 54, atypical meningiomas-WHO grade II, n = 28 and anaplastic meningiomas-WHO grade III, n = 24) were collected in this study.

**Table 1:** Correlation Between RLIP76 Immunoreactivity and Clinicopathologic Characteristics of Meningiomas Patients.

Characteristics	N (%)	RLIP76 expression		P-Value
		Low	High	
**Age, years**				0.529
<60	59 (55.7)	35	24	
≥60	47 (44.3)	25	22	
**Gender**				0.665
Male	44 (41.5)	26	18	
Female	62 (58.5)	34	28	
**MTD, cm**				0.267
<4	48 (45.3)	30	18	
≥4	58 (54.7)	30	28	
**Tumor Location**				0.161
Convexity	44 (41.5)	31	13	
Parasagittal sinus	24 (22.6)	8	16	
Parafalcine	14 (13.2)	7	7	
Skull base	24 (22.6)	14	10	
**Extent of resection**				0.087
Simpson grade I	51 (48.1)	24	27	
Simpson grade II	42 (39.6)	28	14	
Simpson grade III	13 (12.3)	8	5	
**Pathological Classification**				0.008*
WHO I	54 (50.9)	35	19	
WHO II	28 (26.4)	19	9	
WHO III	24 (22.7)	6	18	
**Recurrence**				<0.001*
Yes	40 (37.7)	10	30	
No	66 (62.3)	50	16	

**Abbreviations: MTD, mean tumor diameter**

*P<0.05 was considered statistically significant.

### Cell culture

The human embryonic kidney cell line HEK293 was obtained from the Chinese Academy of Sciences(Shanghai, China) and the malignant meningioma cell lines IOMM-Lee and CH157-MN were obtained from Changzheng hospital(Shanghai, China) [19]. All cell lines were cultured in DMEM supplemented with 8% fetal bovine serum (FBS), penicillin G(100 U/mL), and streptomycin(100 μg/ml) and maintained in monolayer culture at 37°C in humidified air with 5% CO_2_. Viability of the cells was monitored with trypan blue staining.

### Constructs and transfection

The synthetic interfering RNA (siRNA) sequences of RLIP76 were cloned into pLVTHM vectors as described previously and purity was confirmed by Western blot analyses (17, 20). Delivery for RLIP76-siRNA had been established previously [20]. The IOMM-Lee and CH157-MN cells were stably transfected with GFP-siRNA, or RLIP76-siRNA according to the manufacturer's protocol (Invitrogen). Four IOMM-Lee and CH157-MN lines were isolated, one expressing GFP, and another expressing the RLIP76-specific siRNA that led to stable RLIP76 inhibition. Silencing of target gene expression was evaluated 3 d following transfection and did not decrease significantly by 9 d after transfection as determined by Western blot analysis.

### Immunohistochemistry and expression analysis

Human classical meningiomas (WHO grade I, n = 54), atypical meningiomas (WHO grade II, n = 28) and anaplastic meningiomas (WHO grade III n = 24) were provided by the Department of Neuropathology, Institute of Pathology, Changzheng Hospital and PLA NO.322 hospital. The immunohistochemistry was performed as previously described [17]. Briefly, Formalin-fixed, paraffin-embedded, 3-μm human tissue sections were incubated in nonimmune serum for 30 min and then incubated overnight at 4°C in RLIP76 primary monoclonal antibody(1:500; Abcam, ab56815) or Ki-67 primary antibody(1:75; Dako, Glostrup, Denmark). The staining intensity and percentage scores were measured as described [17, 20]. For statistical analysis, RLIP76 expression was divided into “high” (++ and +++) vs. “low” (+ and-). Two independent pathologists examined 5 random fields (1 field = 0.159 mm2 at ×100 magnification) in each sample and scored each sample without knowing patient outcomes (double-blinded).

### Quantitative RT-PCR analysis

Meningioma tissue specimens were stored frozen at -75°C until use. Total RNA were extracted from frozen tissue specimens and glioma cell lines using the RNeasy mini kit (Qiagen). The forward and reverse primers designed for RLIP76 amplification were previously described [17]. The PCR conditions were 5 min at 95°C followed by 40 cycles of 95°C for 30 s, 55°C for 30 s, and 72°C for 30 s. The amplification and melting curves for for RLIP76 andβ-actin are illustrated in S1 Fig. The calculation of Ct for real-time RT-PCR is illustrated in S1 Table.

### Cell proliferation assay

Cell growth was determined by the 3-(4, 5-dimethylthiazol-2-yl)-2, 5- diphenyltetrazolium bromide(MTT) colorimetric cell viability assay as previously described [17]. The number of untransfected IOMM-Lee and CH157-MN cell lines and the stably transfected lines were determined by MTT assays at daily intervals (24, 48, 72, 96, and 120 h after plating).

### Clonogenic assay

Methods for clonogenic assay were described previously [21]. Briefly, 48h after transfection, 1000 cells in 12-well plates were kept in complete medium for 3 weeks. Only colonies containing >50 cells were scored.

### Apoptosis assay

The apoptosis assay was performed as reported previously [17]. Apoptosis was measured using an Annexin V/fluorescein isothiocyanate(FITC) apoptosis detection kit(Bender Med System, CA).

### Statistics

Kaplan-Meier survival analysis was used to compare overall survival time in meningioma patients. The Kruskal—Wallis test was used to analyze the RLIP76 expression and clinicopathological characteristics. The correlation between RLIP76 and Ki-67 immunoreactivity was examined by Spearman's correlation coefficient. Univariate survival analysis was performed using the Kaplan—Meier method and analyzed by the log-rank test to assess survival differences between groups. The Cox proportional hazards model for multivariate survival analysis was used to assess predictors of survival. A two-tailed P value of less than 0.05 was considered statistically significant. Analyses were performed using the SPSS 10.0 statistical software for Windows (SPSS, USA).

## Results

### RLIP76 expression in meningiomas

**Fig 1:**
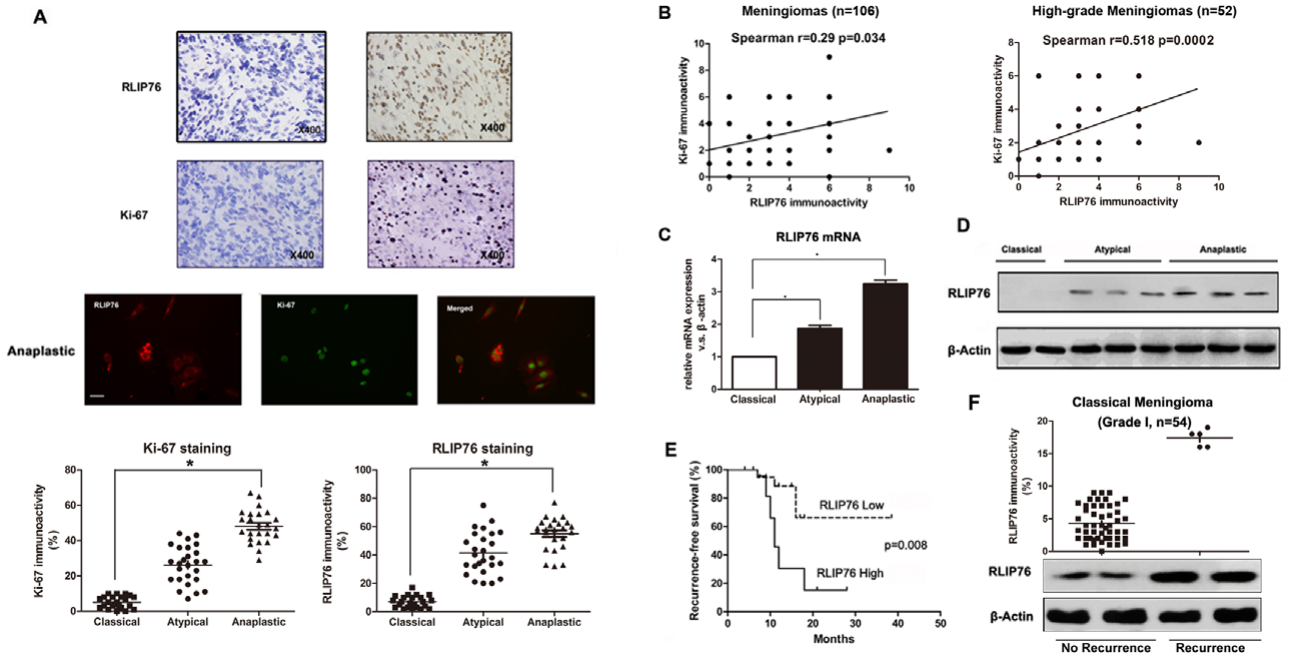
Expression and prognostic value of RLIP76 in human meningiomas. (A) Top: Immunohistochemical expression of RLIP76, and Ki-67 in meningioma of high and low grade. Few RLIP76-positive tumor cells are found in low grade meningiomas(WHO grade I), while strong staining intensity is detected in high grade meningiomas(WHO grade II and III). Middle: Co-localization of RLIP76(red) with Ki-67(green) in glioma cells as revealed by double immunofluorescence labeling. Scale bar, 10 μm. Bottom: RLIP76 and Ki-67 immunoreactivity in meningiomas correlated positively with tumor grade. Statistical analysis revealed a significant increase in RLIP76 and Ki-67 immunoreactivity in high grade meningiomas(Anaplastic/atypical meningiomas) versus low grade meningiomas(classical meningiomas). (B) Left: Positive correlation between RLIP76 and Ki-67 immunolabeling scores in meningiomas. r = 0.29, *p* = 0.034 by Spearman's correlation coefficient. Right: Positive correlation between RLIP76 and Ki-67 immunolabeling scores in malignant meningiomas. r = 0.518, *p* = 0.0002 by Spearman's correlation coefficient. (C) mRNA levels of RLIP76 measured in meningiomas by quantitative real-time PCR (*, *P*<0.05, **, *P*<0.001). (D) Expression of RLIP76 proteins in classical (lanes 1 to 2), atypical(lanes 3 to 5) and anaplastic meningiomas(lanes 6 to 8) measured by Western blot. (E) Kaplan-Meier survival curves according to RLIP76 expression. Patient tumors were analyzed by immunohistochemistry for RLIP76 expression and divided into high and low expression groups as described in Materials and Methods(*P* = 0.008). (F) The dot blot for RLIP76 expression in classical meningiomas (grade I) revealed that those tumors with recurrence had high RLIP76 expression. Western-blot analysis confirmed the similar results.

To validate the significance of differential expression of RLIP76 and Ki-67, a total of 106 meningioma samples were immunostained with RLIP76 and Ki-67 antibodies and quantified by microscopy under a 400X high power field(HPF) system (illustrated in Fig 1A). The demographic and clinical characteristics of studied patients were shown in Table 1. Analysis of immunohistochemical staining intensity revealed high RLIP76 expression in 46 patients(46/106, 43.4%) and low RLIP76 expression in 60 patients(60/106, 56.6%). Statistical analysis showed increased RLIP76 immunostaining score in anaplastic atypical meningiomas versus classical meningiomas(5.48± 1.02 *vs*. 1.26±0.85, *P*<0.001). High-grade meningioma showed great immunoreactivity for RLIP76 and Ki-67 proteins by double immunofluorenscence labeling (Fig 1A). Spearman correlation of RLIP76 and Ki67 at r = 0.29 was low in 106 meningiomas because Ki67 expression was extremely variable in low-grade meingiomas. We then did a Spearman plot of RLIP76 and Ki-67 in 52 high-grade meningiomas(atypical meningiomas-WHO grade II, n = 28 and anaplastic meningiomas-WHO grade III, n = 24) to further explore the relation between RLIP76 and Ki-67 expression. We found that immunostaining levels of RLIP76 and Ki-67 were positively correlated in atypical and anaplastic meningiomas (Fig 1B). Additional validation by the real-time PCR confirmed the increase of RLIP76 mRNA expression in anaplastic meningiomas and atypical meningiomas compared to classical meningiomas (Fig 1C). We also examined the level of RLIP76 protein by Western analysis. As shown in Fig 1D, anaplastic meningiomas and atypical meningiomas expressed detectable and comparable levels of RLIP76, whereas all of the classical meningiomas did not express detectable levels of RLIP76 protein. For grade I meningioma, high RLIP76 expression was observed in those with recurrence, while benign tumors had low RLIP76 expression, suggesting that RLIP76 expression may induce more aggressive tumor behavior (Fig 1F). Thus, we conclude that RLIP76 expression correlated with tumor progression and with proliferating meningiomas.

### RLIP76 Expression and Clinicopathologic Features

To determine whether RLIP76 expression was correlated with clinicopathological parameters, we investigated the association between RLIP76 expression and histological grading, recurrence and other clinical features of 106 patients with meningiomas(Table 1). Positive correlations were found between the RLIP76 labeling index and higher histological grade of the meningiomas (*P*<0.05). Furthermore, RLIP76 was significantly overexpressed in meningioma patients with recurrence (*P*<0.001), while no significant associations were found between RLIP76 expression and age, sex, tumor size, and tumor location. Next, we used univariate analysis to further explore the correlation between recurrence rate and RLIP76 expression. Consistent with previous Kruskal—Wallis test (Table 1), univariate survival analysis revealed that meningioma patients with high RLIP76 expression would more likely have tumor recurrence as compared with those with low RLIP76 expression(*P* = 0.007, Table 2). In addition, expression of Ki-67(*P* = 0.001) and histological grade (*P*<0.01, Table 2) also had a positive relationship with recurrence. There were no significant correlations between recurrence rate and other clinical features. Thus, we concluded that RLIP76 expression was a significant risk factor for malignant recurrence.

**Table 2:** Univariate Analysis of the Associations Between Prognostic Variables and Recurrence in 106 Meningiomas Patients.

Characteristics	N	Recurrence rate (%)	P-values
**Pathological Classification**			0.000*
WHO I	54	9 of 54 (16.7)	
WHO II	28	9 of 28 (32.1)	
WHO III	22	22 of 24 (91.7)	
**Ki-67**			0.001*
Low	54	6 of 54 (11.1)	
High	52	34 of 52 (60.9)	
**RLIP76**			0.007
Low	60	10 of 60 (16.7)	
High	46	30 of 46 (65.4)	
**Extent of resection**			0.07
Simpson grade I	51	21 of 51 (41.2)	
Simpson grade II	42	12 of 42 (28.6)	
Simpson grade III	13	7 of 13 (53.8)	
**MTD, cm**			0.092
<4	48	14 of 48 (29.2)	
≥4	58	26 of 58 (44.8)	
**Tumor Location**			0.405
Convexity	44	10 of 44 (22.7)	
Parasagittal sinus	24	12 of 24 (50)	
Parafalcine	14	4 of 14 (28.6)	
Skull base	24	14 of 24 (58.3)	
**Age, years**			
<60	59	23 of 59 (39)	0.102
≥60	47	17 of 47 (36.2)	
**Gender**			0.528
Male	44	16 of 44 (36.4)	
Female	62	24 of 62 (38.7)	

* P<0.05 was considered statistically significant.

### RLIP76 Immunoreactivity and Recurrence-Free Survival Rate

As classical meningioma(WHO grade I) are considered benign with low recurrence, we did a Kaplan Meier plot of RLIP76 high/low and recurrence in 52 high-grade meningiomas(atypical meningiomas-WHO grade II, n = 28 and anaplastic meningiomas-WHO grade III, n = 24) by comparing with all 54 benign tumors(classical meningiomas- WHO grade I) to make recurrence-free survival analysis more convincing. As predicted, recurrence-free survival rate was higher in malignant meningioma patients with lower RLIP76 expression (Fig 1E). Multivariate analysis revealed that high RLIP76 expression(staining score≥4) at diagnosis(*P* = 0.002) and pathological grade(*P* = 0.000, Table 3) were independent prognostic factors for patients with meningioma. These results suggest that RLIP76 expression may be an independent factor for recurrence-free survival.

**Table 3:** Multivariate Analysis of Potential Factors Affecting Recurrence-Free Survival in 106 Meningiomas Patients.

Variable	B	SE	Wald	df	Sig.	HR	95.0% CI for HR	
							Lower	Upper
WHO Grade	0.824	0.235	12.231	1	0.000*	2.279	1.436	3.615
RLIP76	1.187	0.379	9.808	1	0.002*	3.277	1.559	6.888
Ki-67	1.393	0.458	9.251	1	0.002*	4.026	1.641	9.877

**B, regression coefficient; Wald, wald statistics; df, degree of freedom; HR, hazard rate**

* P<0.05 was considered statistically significant.

### Knockdown of RLIP76 expression reduces the proliferation of meningioma cells in vitro

**Fig 2:**
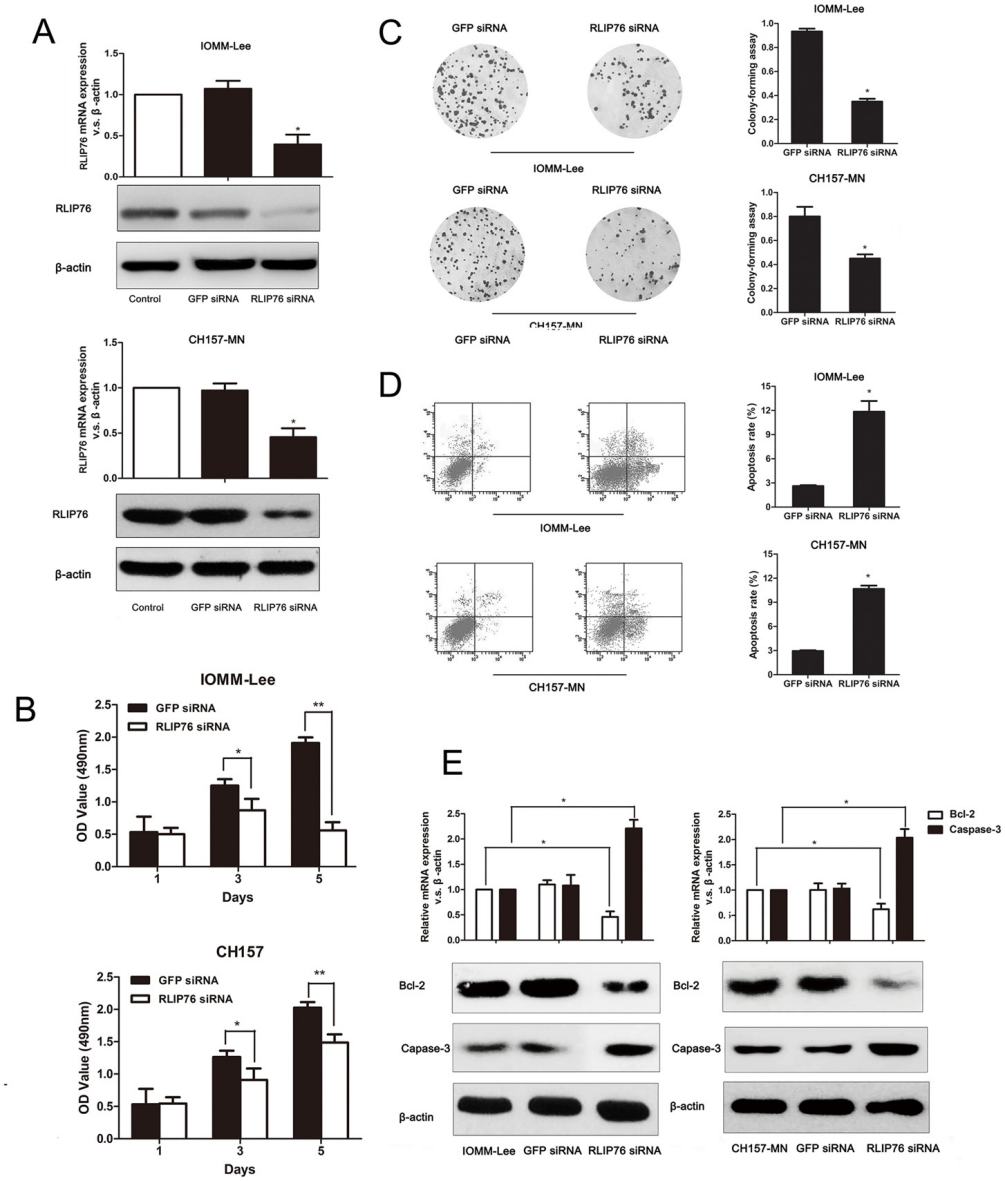
Effect of RLIP76 expression on cell proliferation, colony formation and apoptosis in meningiomas cell lines. (A) PCR and Western blot analysis revealed reduced RLIP76 mRNA and protein in IOMM-Lee and CH157-MN cells transfected with the RLIP76-targeted siRNA compared to GFP-transfected and control cells. (B) Effect of RLIP76 knockdown on IOMM-Lee and CH157-MN cell proliferation as measured by the MTT cell viability assay at different times after cell plating (*, *P*<0.05). (C) Effect of RLIP76 knockdown on IOMM-Lee and CH157-MN proliferation as measured by clonogenic assay (*, *P*<0.05). (D) The number of apoptotic cells was significantly higher in IOMM-Lee and CH157-MNcells transfected with RLIP76-siRNA as measured by PI staining and flow cytometry(*, *P*<0.05). (E) PCR and Western-blots revealed that caspase-3 mRNA and protein levels were significantly higher while Bcl-2 mRNA and protein expression was markedly lower in IOMM-Lee and CH157-MN cells transfected with RLIP76 siRNA.

Stable transfection of IOMM-Lee and CH157-MN cell lines with lentivirus-based RLIP76 siRNA dramatically decreased the RLIP76 expression at both mRNA and protein level (Fig 2A). Consistent with an important role of RLIP76 in mengioma patients survival, knockdown of RLIP76 expression in IOMM-Lee and CH157-MN cell lines suppressed the growth of both IOMM-Lee and CH157-MN cells by MTT assays (Fig 2B) and reduced the cell proliferation as evidenced by clonogenic assays (Fig 2C).

### RLIP76 knockdown increases apoptosis of meningioma cells in vitro

To determine whether RLIP76 affected cell apoptosis, we used flow cytometric analysis to examine apoptosis in these cell lines and found that enhanced apoptosis induced in siRNA-transfected IOMM-LEE and CH157-MN cells compared to GFP-transfected cells (Fig 2D). Real-time PCR revealed that knockdown of RLIP76 led to a significant decrease of anti-apoptotic protein Bcl-2 in IOMM-LEE and CH157-MN cells compared to control cells, while the expression of pro-apoptotic effector caspase-3 mRNA was significantly higher (Fig 2E, upper portion). In parallel, the efficiency of silencing RLIP76 was measured by Western blot (Fig 2E, lower portion). Thus, these results demonstrated that knockdown of RLIP76 expression induced apoptosis by down-regulating Bcl-2 and up-regulating Caspase-3 in IOMM-LEE and CH157-MN cells.

## Discussion

In recent years, exciting development has been made in the research on molecular genetics of malignant meningiomas. The resulting information has led the way for an increasing interest in potential genetics-based treatments [4]. In this study, we found that RLIP76 expression in human meningioma was associated with the pathological grade, with the highest level of expression in anaplastic meningiomas(WHO grade III) and lowest expression in classical meningiomas(WHO grade I). Moreover, we found a strong positive correlation between RLIP76 expression and the proliferation marker Ki-67 in 106 meningioma tumors, suggesting that RLIP76 overexpression led to a highly proliferate phenotype. In addition, the expression of RLIP76 was correlated with the recurrence rate of meningioma patients, and higher RLIP76 expression was associated with shorter recurrence-free survival. Since RLIP76 expression was associated with higher grade tumors by association, it should also be associated with increased recurrence. In order to avoid this bias, we made the recurrence-free survival analysis by histological types, for example taking out all benign tumors and making a Kaplan Meier plot of RLIP76 expression and recurrence to make the analysis more convincing. As predicted, Cox regression analysis revealed that RLIP76 was actually an independent factor for recurrence-free survival in malignant meningiomas. Results from this study showed that RLIP76 protein expression was positively correlated with the pathological stages and recurrence of meningiomas.

Emerging evidences show that altered apoptosis is the most common biological abnormalities found in meningiomas. Recently, a large number of studies have shown RLIP76 plays a requisite role in diverse cellular functions including apoptosis, and is overexpressed in a variety of malignancies [13, 14, 16, 17, 22, 23]. In our study, we demonstrated that RLIP76 was also an important mediator of malignant meningiomas. We found that down-regulation of RLIP76 expression decreased meningioma proliferation in part by increasing apoptosis, consistent with previous studies demonstrating that increased RLIP76 expression was related with higher proliferation in malignant tumors. Furthermore, to ascertain the mechanisms of apoptosis induced by the RLIP76-targeted siRNA, we measured the expression of Bcl-2 and caspase-3 mRNAs and proteins by real-time PCR and Western blotting. Knock- down of RLIP76 decreased Bcl-2 expression and increased caspase-3 expression at both the mRNA and protein levels, implying a functional interaction between RLIP76 and the Bcl-2 and caspase-3 pathways in meningiomas.

RLIP76 produces oncogenic action by regulating apoptosis signaling in human cancer cells. High expression of RLIP76 decreases apoptosis levels through interactions with a spectrum of functionally distinct proteins [13, 14, 24–26]. It has been reported that RLIP76-related Caspase-3 and Bcl-2 are overexpressed in high grade meningioma, which correlated with recurrence and prognosis in meningioma [27, 28]. RLIP76 is also identified as a Ral effector protein by linking Ral GTPase to Rho pathway [29]. RLIP76 binds to Ral and triggers a GAP activity on cdc42, a member of the small Rho GTPases [30]. It is noteworthy that Rho pathway is thought to be an essential step in activation of phosphorylation cascades which stimulate meningioma cell proliferation and apoptosis [31]. Thus, it is reasonable to speculate that RLIP76 could modulate multiple cellular signaling pathways, particularly classical apoptosis pathway and Rho pathway, and by doing so, ultimately contributes to cancer development. Given the importance of RLIP76 in classical apoptosis pathway and Rho signaling, it is also reasonable to speculate that agents specifically targeting RLIP76 could be beneficial in malignant meningioma patients.

In conclusion, we demonstrated that high levels of RLIP76 expression were associated with unfavorable outcomes in meningioma patients. These findings imply that RLIP76 expression level is a possible indicator in predicting the recurrence of meningiomas, and it is possible that certain agents specifically target on RLIP76 could be used in patients with high expression of RLIP76 in future.

## Supporting Information

S1 Fig Amplification curves for RLIP76 (A) and β-actin (C). Melting curves for RLIP76 (B) and β-actin (D). (TIF)

S1 Table The calculation of Ct for real-time RT-PCR. (DOC)
