# Supplementary figures and images for: Overexpression of RLIP76 Required for Proliferation in Meningioma Is Associated with Recurrence
Source: PLoS One. 2015 May 20;10(5):e0125661. doi: 10.1371/journal.pone.0125661 (PMC4439061; doi:10.1371/journal.pone.0125661)

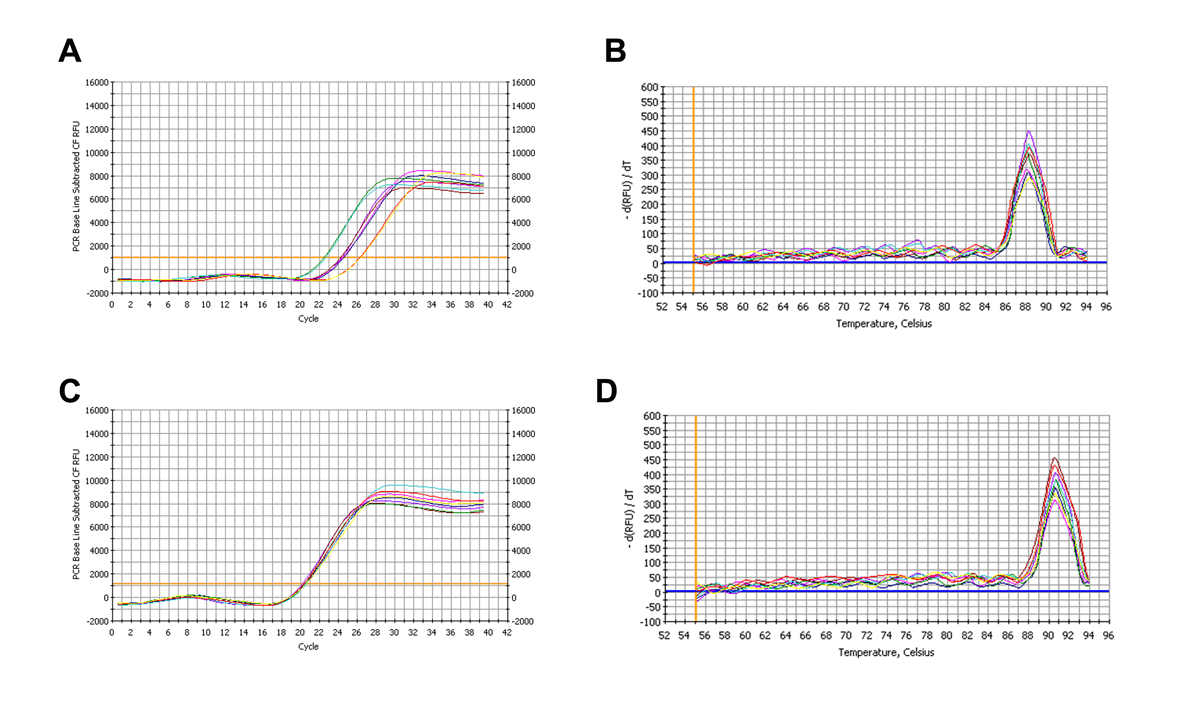

Supplement: S1 Fig — (TIF) [file pone.0125661.s001.tif]
