# Supplementary material for: Overexpression of RLIP76 Required for Proliferation in Meningioma Is Associated with Recurrence
Source: PLoS One. 2015 May 20;10(5):e0125661. doi: 10.1371/journal.pone.0125661 (PMC4439061; doi:10.1371/journal.pone.0125661)
Supplement: S1 Table — (DOC) [file pone.0125661.s002.doc]

**S1 Table. The calculation of Ct for real-time RT-PCR.**

|  |  |  |  |  |  |  |  | graph |  | graph |  | graph |
| --- | --- | --- | --- | --- | --- | --- | --- | --- | --- | --- | --- | --- |
|  |  |  |  |  |  |  |  | ratio |  | error pos |  | error neg |
| sample | β-actin | sd | RLIP76 | sd | ΔCt | sd ΔCt | delta ΔCt | 2^(-ΔCt) | 2^(-ΔCt+sd) | sd pos | 2^(-ΔCt-sd) | sd neg |
| Classical | 20.550 | 0.071 | 24.950 | 0.071 | 4.400 | 0.100 | 0.000 | 1.000 | 1.072 | 0.072 | 0.933 | 0.067 |
| Atypical | 20.450 | 0.071 | 24.150 | 0.071 | 3.700 | 0.100 | -0.700 | 1.625 | 1.741 | 0.117 | 1.516 | 0.109 |
| Anaplastic | 20.350 | 0.071 | 23.050 | 0.212 | 2.700 | 0.224 | -1.700 | 3.249 | 3.794 | 0.545 | 2.783 | 0.466 |
